# Persistently raised inflammatory markers in a patient with resolved lower respiratory tract infection: a diagnostic challenge

**DOI:** 10.1093/omcr/omag073

**Published:** 2026-05-24

**Authors:** Khalid Khan, Housameddin Ghazzawi

**Affiliations:** General Medicine, Lincoln County Hospital, United Lincolnshire Teaching Hospitals NHS Trust, Greetwell Road, Lincoln, Lincolnshire, LN2 5QY, United Kingdom; Acute Medicine, Pilgrim Hospital Boston, United Lincolnshire Teaching Hospitals NHS Trust, Sibsey Road, Boston, Lincolnshire, PE21 9QS, United Kingdom

**Keywords:** persistently raised inflammatory markers, post-infectious immune dysregulation, inflammaging, neutrophilic leucocytosis, asymptomatic elderly patient, diagnostic uncertainty

## Abstract

We present the case of a woman in her early nineties who was admitted after recurrent falls and a short history of respiratory symptoms. She was diagnosed with influenza A lower respiratory tract infection (LRTI) complicated by hospital-acquired pneumonia (HAP). Despite clinical and radiological recovery, inflammatory markers remained persistently elevated, including neutrophilic leucocytosis, thrombocytosis, and elevated C-reactive protein (CRP), without residual symptoms or objective evidence of infection. Extensive investigation and multidisciplinary input failed to identify an infective, autoimmune, or malignant cause. This case illustrates the challenge of interpreting persistent biochemical abnormalities in older adults, highlights the importance of clinical context, and cautions against over-investigation and unnecessary antibiotic use in asymptomatic patients.

## Introduction

Persistent elevation of inflammatory markers after resolution of infection presents a diagnostic dilemma, especially in older adults. Age-related immune dysregulation, including immunosenescence and chronic low-grade systemic inflammation (‘inflammaging’), may contribute to sustained biochemical abnormalities in the absence of active disease [1]. Consequently, persistently elevated inflammatory markers in older adults may reflect biological ageing rather than pathology, complicating interpretation.

Most published reports addressing persistently raised inflammatory markers describe patients who are symptomatic, systemically unwell, or found to have an infective, autoimmune, or malignant process. Literature describing clinically stable, asymptomatic elderly individuals with persistently elevated inflammatory markers despite negative investigations is limited. This case documents an extensive multidisciplinary workup in a woman in her early nineties without residual symptoms or objective evidence of infection, highlighting the importance of interpreting abnormal results within clinical context to avoid over-investigation and overtreatment.

## Case report

A woman in her early nineties was admitted after recurrent falls and a short history of cough, breathlessness, and wheeze. She lived at home with family support for instrumental activities, remained independent in basic activities, and mobilised with a frame, consistent with Clinical Frailty Scale (CFS) score 5 (mild frailty). Her past medical history included hypertension, atrial fibrillation, glaucoma, gallstones, chronic gastric ulcer disease, degenerative disc disease, and ductal carcinoma in situ of the right breast treated with surgery and radiotherapy in 1992 without recurrence.

**Figure 1:**
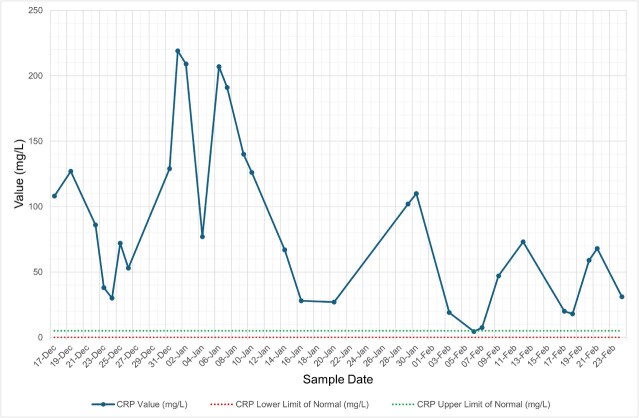
Graph illustrating serial trends in C-reactive protein (CRP) from December 2024 to February 2025. The values remained persistently elevated above the upper limit of normal, including during clinical recovery. Green dashed line = upper limit of normal; red dashed line = lower limit of normal.

**Figure 2:**
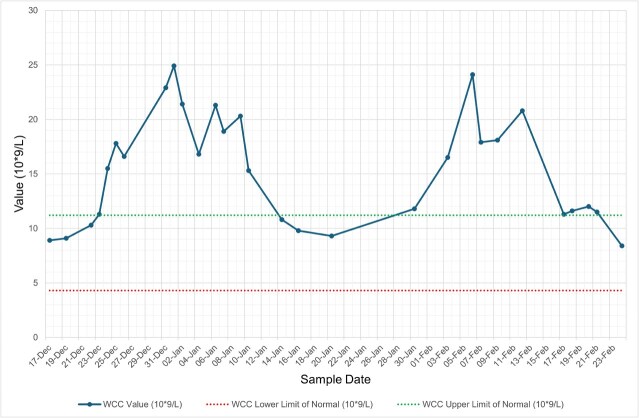
Graph illustrating serial trends in white cell count (WCC) from December 2024 to February 2025. WCC remained elevated above the upper limit of normal throughout most of the admission, with delayed normalisation evident post-clinical recovery of the patient. Green dashed line = upper limit of normal; red dashed line = lower limit of normal.

**Figure 3:**
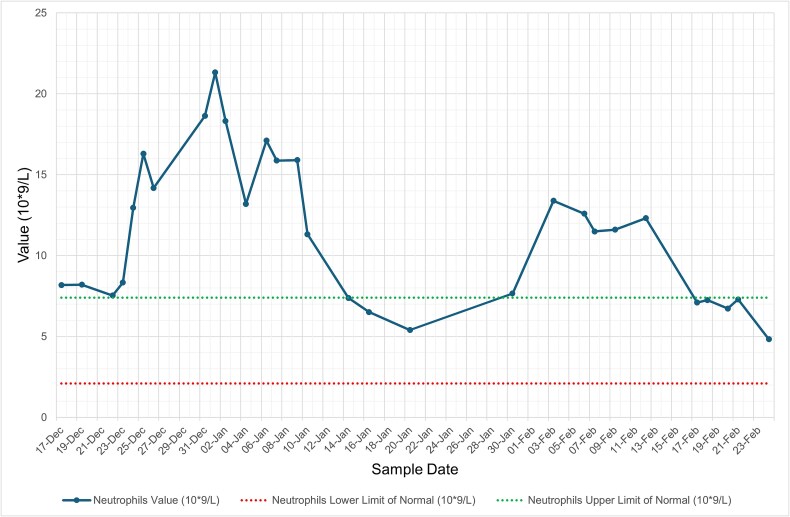
Graph illustrating serial trends in neutrophils from December 2024 to February 2025. Neutrophilia persisted throughout most of the admission closely following the trend in WCC. The abnormally elevated levels finally trailed off post-clinical recovery as with WCC. Green dashed line = upper limit of normal; red dashed line = lower limit of normal.

**Figure 4:**
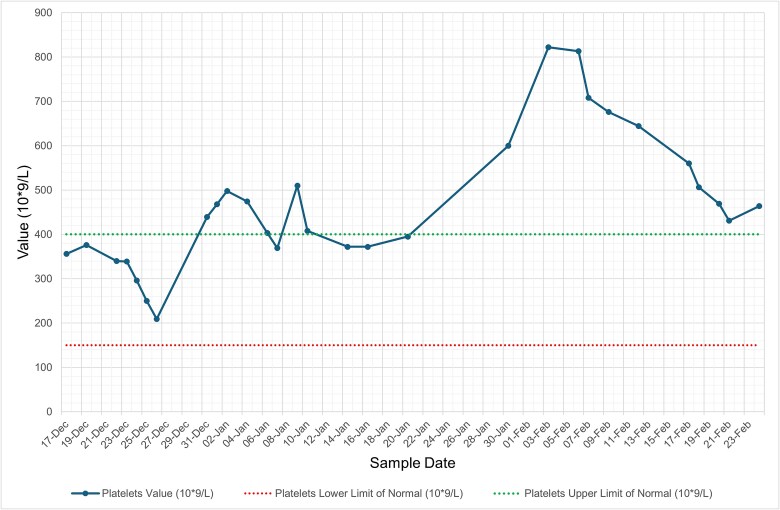
Graph illustrating serial trends in platelets from December 2024 to February 2025. Interestingly, values peaked and remained abnormally raised during the latter half of the patient’s admission well into their clinical recovery. Green dashed line = upper limit of normal; red dashed line = lower limit of normal.

She was diagnosed with influenza A lower respiratory tract infection (LRTI) complicated by hospital-acquired pneumonia (HAP) and was managed with oseltamivir and levofloxacin (penicillin allergy), with resolution of respiratory symptoms and radiological changes. Despite being afebrile, asymptomatic, and haemodynamically stable, her inflammatory markers remained persistently abnormal, with neutrophilic leucocytosis, thrombocytosis, and elevated C-reactive protein (CRP) (Figs 1–4, and Table 1). Repeat cultures and imaging remained unremarkable, and autoimmune screen was negative (Tables 1–3).

**Table 1:** Table showing persistent laboratory abnormalities after clinical recovery late in admission.

Test	Value	Normal range
C-Reactive Protein	▲ 68	*0–5 mg/l*
Platelets	▲ 431	*150–400* × *10^9/l*
White Cell Count	▲ 11.5	*4.3–11.2* × *10^9/l*
Lymphocytes	2.54	*1.0–3.6* × *10^9/l*
Neutrophils	7.29	*2.1–7.4* × *10^9/l*
Monocytes	▲ 1.04	*0.3–1.0* × *10^9/l*
Eosinophils	0.47	*0.02–0.5* × *10^9/l*
Basophils	▲ 0.12	*0.02–0.1* × *10^9/l*
Myeloperoxidase Ab	<3.2	*0–20 U*
Proteinase-3 Ab	<2.3	*0–20 U*
Double-stranded DNA Ab	<9.8	*0–24 IU/ml*
Erythrocyte Sedimentation Rate	▲ 67	*9–20 mm/h*
Anti-nuclear Antibody (ANA)	Negative	
Smooth Muscle Antibody	Negative	
Gastric Parietal Cell Antibody	Negative	
Mitochondrial Antibody	Negative	
Liver Kidney Microsomal Antibody	Negative	
Rheumatoid Factor	▲ 15	*0–14 IU/ml*
C3	1.69	*0.90–1.80 g/l*
C4	▲ 0.44	*0.10–0.40 g/l*
CCP Antibodies	8	*0–17 U/ml*
Angiotensin Converting Enzyme	▲ 76	*20–70 U/l*

**Table 2:** Microbiological results of specimens collected between December 2024 and February 2025.

Specimen	Date	Result	Organism	Antibiotic sensitivity
Nasopharyngeal swab	17/12/2024	Positive	Influenza A	*N/A*
Blood culture	17/12/2024	Negative	-	-
Blood culture	02/01/2025	Negative	-	-
Urine culture	02/01/2025	*Not indicated—normal WCC/bacterial count*	-	-
Blood culture	07/01/2025	Negative	-	-
Blood culture	10/01/2025	Negative	-	-
Blood culture	03/02/2025	Negative	-	-
Urine culture	06/02/2025	*Not indicated—normal WCC/bacterial count*	-	-

**Table 3:** Summary of imaging investigations and incidental findings during admission.

Modality	Date	Report summary
CXR	31/12/2024	‘The lungs are clear, no focal collapse, air space opacification, pleural effusion or evidence of pneumothorax identified.’
CTPA	22/12/2024	‘Subsegmental consolidation, predominantly in the right lower lobe—likely related to infection.’
CXR	04/01/2025	‘Subtle patchy airspace opacification in the lower zones is suggestive of ongoing infection. No focal lung lesion, gross pleural effusion or pneumothorax.’
CXR	07/01/2025	‘The lungs are clear, no focal collapse, air space opacification, pleural effusion or evidence of pneumothorax identified.’
CTCAP	12/01/2025	‘Patchy air space opacities seen in both lower lobes, likely infective. Left adrenal nodule. Infrarenal aortic aneurysm. Atherosclerotic Penetrating ulcer seen in aortic arch.’
CTA	16/01/2025	‘Penetrating ulcer is arising from the aortic arch. Aneurysm of the infrarenal abdominal aorta.’
CXR	30/01/2025	‘No focal lung lesion, confluent airspace opacification, pleural effusion or pneumothorax.’
USS Liver	11/02/2025	‘The gallbladder contains calculi and biliary sludge. The CBD and liver appear normal. Gallstone with biliary sludge; no cholecystitis.’
CXR	28/02/2025	‘No focal lung lesion, confluent airspace opacification, pleural effusion or pneumothorax. No interval change since the previous examination of 30th January 2025.’
PET Scan	07/03/2025	‘Dynamic changes in both lungs, which may be inflammatory or infective, warrant future review with CT. Separate from this, no definite site of inflammatory or infective etiology is identified, nor any FDG-avid malignancy.’

Computed tomography chest-abdomen-pelvis (CTCAP) showed no infective focus but identified an incidental penetrating aortic arch ulcer and infra-renal abdominal aortic aneurysm. Computed tomography angiography (CTA) of aorta reviewed by vascular surgery confirmed chronic atherosclerotic changes unrelated to infection. Although atherosclerosis may contribute to low-grade systemic inflammation, no features of active vascular inflammation were identified. A left adrenal nodule was characterised as a benign adenoma following endocrinology and radiology MDT review.

Given radiological findings of peri-hilar granulomas and lower-lobe ground-glass changes, respiratory input was sought to explore alternative inflammatory or interstitial pathology, but the findings were attributed to resolving LRTI instead of a separate inflammatory process and follow-up chest imaging in 6 weeks was recommended. Abdominal ultrasound for abnormal liver enzymes showed gallstones and biliary sludge without cholecystitis, deemed incidental by general surgery.

Repeated haematology reviews concluded the abnormalities represented a reactive process rather than a myeloproliferative disorder. Microbiology advised against further antibiotic escalation given repeated negative cultures and recommended a positron emission tomography (PET) scan to exclude occult infection. This demonstrated improving residual lung changes but no infection, malignancy, or vasculitis, and confirmed incidental gallstones in a non-inflamed gallbladder, benign left adrenal adenoma, and degenerative skeletal changes.

Although respiratory symptoms had resolved, discharge to transitional care was delayed because the receiving community team questioned her raised inflammatory markers. After reassurance that these reflected post-infective immune dysregulation rather than active infection, she was safely transferred.

The patient had an advanced care plan, including a decision not to attempt cardiopulmonary resuscitation, recommending ward-based care and supporting the collective decision to prioritise rehabilitation over definitive diagnosis.

She did not return to her pre-admission functional baseline and was discharged to a nursing home after rehabilitation. Her confirmed stable incidental findings did not require intervention, and surveillance was not recommended given her age and frailty.

## Discussion

This case illustrates the difficulty of distinguishing residual inflammation from active pathology in older adults. While inflammatory markers guide clinical decision-making, their persistence in asymptomatic patients requires careful clinical interpretation.

A literature review using the DiCenso 6S pyramid identified limited guidance on this scenario (Table 4). Corrales-Medina et al. described persistent pulmonary inflammation after pneumonia, though in symptomatic individuals [2]. Other reports focus on post-acute infection syndromes, often COVID-related (Choutka et al.; Behzadi et al.; Yao et al.) [3–5]. Puchta et al. proposed TNF-mediated monocyte dysfunction as a mechanism for inflammaging [6], while Korn et al. reported unexplained inflammatory markers with radiological abnormalities [7].

**Table 4:** Summary of results from a structured literature search conducted to identify previously reported cases relevant to the present report.

Source	DiCenso 6S pyramid level	Key findings & clinical implications
Choutka J, Jansari V, Hornig M, and Iwasaki A. Unexplained Post-Acute Infection Syndromes. Nature Medicine.	Synopses of Syntheses (Narrative Review)	Provides a synthesis of post-acute infection syndromes (including MIS-A and post-COVID inflammation), highlighting diagnostic complexity and immune dysregulation mechanisms.
Behzadi F, Ulloa NA, and Danckers M. Multisystem Inflammatory Syndrome in Adults: A Case Report and Review of the Literature.	Summaries (Case-based Literature Review)	Summarises multiple MIS-A case reports with clinical features, diagnostics, and treatment, offering a concise review useful for clinical decision-making.
Corrales-Medina VF *et al*. Persistent Lung Inflammation after Clinical Resolution of Community-acquired Pneumonia as Measured by ^18^FDG-PET/CT Imaging.	Studies (Primary Observational Study)	~68% of elderly CAP survivors with persistent pulmonary inflammation on ^18^FDG-PET/CT 30–45 days post-recovery (n = 22).
Puchta A *et al.* TNF Drives Monocyte Dysfunction with Age and Results in Impaired Anti-pneumococcal Immunity.	Studies (Translational Study)	Puchta et al. found age-associated TNF-driven monocyte dysfunction increasing basal inflammation (‘inflammaging’) in elderly.
Korn L, Xu M, and Brunet C. Case Report: Elevated Inflammatory Markers & A Hypermetabolic Mass.	Studies (Case Report – Clinical Website Publication)	Detailed case of persistently raised inflammatory markers with hypermetabolic findings without clear infective or malignant source. Relevant due to diagnostic parallels.
Yao Q, Waley L, and Liou N. Adult Presentation of Multisystem Inflammatory Syndrome (MIS) Associated with Recent COVID-19 Infection: Lessons Learnt in Timely Diagnosis and Management.	Studies (Case Report)	Single-patient case of MIS-A with delayed systemic inflammation post-COVID in a symptomatic patient. Relevant due to similar diagnostic challenge of post-infectious inflammation.

Note: Additional references cited in the main text were not part of the literature search and are included for contextual discussion.

Our structured search did not identify comparable reports of clinically stable, asymptomatic elderly patients with persistently elevated inflammatory markers. Although our patient had chronic comorbidities that could contribute to low-grade inflammation, there was no clinical, radiological, or microbiological evidence of acute disease. Her breast malignancy had been in long-term remission, and gastric ulcer disease showed no active features.

Emerging evidence links raised inflammatory markers with frailty, sarcopenia, and cognitive decline [8–10], suggesting CRP elevation may reflect biological ageing instead of ongoing pathology. In this context, persistent abnormalities may represent baseline vulnerability rather than disease requiring intervention.

Extensive investigations yielded incidental findings without altering management. Differential diagnoses included subclinical or occult infection, chronic inflammatory or autoimmune disease, and post-infectious immune dysregulation. In the context of negative investigations and clinical stability, post-infectious immune dysregulation on a background of vascular and degenerative disease was considered the most plausible explanation.

Importantly, abnormal laboratory values created barriers to discharge, illustrating the risks of over-interpreting isolated biochemical abnormalities in clinically stable older adults. Our patient’s care plan emphasised proportionality of investigations and supported rehabilitation over continued diagnostic escalation. She later re-presented to the emergency department with abnormal inflammatory markers but did not require admission, highlighting the challenges these results posed across care settings and reinforcing their limited clinical significance.

This case adds to the limited literature on persistent inflammation in asymptomatic elderly patients and supports a clinically grounded approach when objective evidence of infection is absent.

### Learning points

1. Persistent inflammatory markers in elderly patients must be interpreted within clinical context
2. Post-infectious immune dysregulation and inflammaging may explain sustained abnormalities
3. Clinical stability and symptom resolution outweigh isolated inflammatory markers when assessing recovery
4. Judicious antibiotic use is essential to avoid harm and resistance
5. Multidisciplinary input supports balanced decision-making
6. Over-investigation may delay discharge without improving outcomes
